# High-yield synthesis and purification of recombinant human GABA transaminase for high-throughput screening assays

**DOI:** 10.1080/14756366.2021.1975697

**Published:** 2021-09-12

**Authors:** Mingu Gordon Park, Ah-reum Han, Su Yeon Kim, Tai Young Kim, Ho Min Kim, C. Justin Lee

**Affiliations:** a KU-KIST Graduate School of Converging Science and Technology, Korea University, Seoul, South Korea; b Center for Cognition and Sociality, Institute for Basic Science (IBS), Daejeon, South Korea; c Center for Biomolecular and Cellular Structure, Institute for Basic Science (IBS), Daejeon, South Korea; d Graduate School of Medical Science & Engineering, Korea Advanced Institute of Science and Technology (KAIST), Daejeon, South Korea

**Keywords:** 4-Aminobutyrate transaminase, isolation and purification, high-throughput screening assays, gabaculine, vigabatrin

## Abstract

Many studies have focussed on modulating the activity of γ-aminobutyric acid transaminase (GABA-T), a GABA-catabolizing enzyme, for treating neurological diseases, such as epilepsy and drug addiction. Nevertheless, human GABA-T synthesis and purification have not been established. Thus, biochemical and drug design studies on GABA-T have been performed by using porcine GABA-T mostly and even bacterial GABA-T. Here we report an optimised protocol for overexpression of 6xHis-tagged human GABA-T in human cells followed by a two-step protein purification. Then, we established an optimised human GABA-T (0.5 U/mg) activity assay. Finally, we compared the difference between human and bacterial GABA-T in sensitivity to two irreversible GABA-T inhibitors, gabaculine and vigabatrin. Human GABA-T in homodimeric form showed 70-fold higher sensitivity to vigabatrin than bacterial GABA-T in multimeric form, indicating the importance of using human GABA-T. In summary, our newly developed protocol can be an important first step in developing more effective human GABA-T modulators.

## Introduction

γ-aminobutyric acid (GABA) is the major inhibitory neurotransmitter synthesised and released from GABAergic neurons and astrocytes in the mammalian central nervous system (CNS)^1–4^. GABA is synthesised by glutamate decarboxylase (GAD; EC 4.1.1.15) in GABAergic neurons and by monoamine oxidase (MAO-B; EC 1.4.3.4) or diamine oxidase (DAO; EC 1.4.3.22) in astrocytes^4–6^. However, the only GABA-catabolizing enzyme in the mammalian CNS is GABA transaminase (GABA-T; EC 2.6.1.19) encoded by the *ABAT* gene^7^. GABA-T catalyses the conversion of GABA to succinic semialdehyde (SSA) concomitantly with the conversion of α-ketoglutarate (α-KG) to glutamate. Subsequently, SSA is oxidised to succinic acid (SA) by the enzyme SSA dehydrogenase (SSADH; EC 1.2.1.24). GABA-T and SSADH are mitochondrial enzymes and exist both in GABAergic neurons and astrocytes^8–10^. Because the transaminase reaction cannot be directly monitored, we can utilise dehydrogenation of SSA from GABA-T reaction to indirectly monitor GABA-T activity by detecting a conversion of NADP^+^ to NADPH^11^.

Therapeutic approaches for a large number of neurological and psychiatric disorders due to excessive excitatory neurotransmission involve elevating brain GABA levels. And the most efficient way to achieve that is to inhibit GABA-T and disrupt the GABA degradation pathway. For example, vigabatrin, CPP-115, and OV329, potent irreversible inhibitors of GABA-T, have been developed for the treatment of epilepsy and addiction^12–14^. There have been numerous studies to extensively purify and characterise native GABA-T from the mammalian brain or peripheral tissues^15–23^. Nevertheless, high-yield human GABA-T synthesis and purification have not been established despite the high demand. Thus, biochemical studies on GABA-T and the development of GABA-T modulators for human use have been carried out mostly by using porcine GABA-T which has 96% identity with human GABA-T^24^. Silverman group especially has designed, synthesised, and evaluated a variety of effective irreversible GABA-T inhibitors with endogenous GABA-T purified from porcine brain^25–27^. There has even been a kinetic study on the inhibition of bacterial GABA-T from *Pseudomonas fluorescens* by vigabatrin and taurine^28^. Unfortunately, the modes of action for reported GABA-T inhibitors have not been studied on human GABA-T. Purifying native human GABA-T from the human brain or peripheral tissues requires not only approval by the Institutional Review Board, but also too much time and expense for the amount of purified native enzymes to be obtained. To overcome these limitations, a few studies performed overexpression of recombinant human, porcine and bovine GABA-T in *Escherichia coli* (*E. coli*)^29–31^. However, these approaches could not provide appropriate post-translational modifications (PTMs) for mammalian GABA-T and resulted in the formation of both insoluble GABA-T and inclusion bodies. Therefore, it is highly necessary to develop a simple, scalable and efficient protocol for high-yield synthesis and purification of human GABA-T with proper folding and PTMs.

In this study, we utilised the Expi293™ expression system, Nickel-Nitrilotriacetic acid (Ni-NTA) chromatography, and size-exclusion chromatography to obtain 6xHis-tagged human GABA-T homodimer. Using the purified human GABA-T, we established an optimised protocol for human GABA-T enzyme activity assay and compared the difference between human and bacterial GABA-T in sensitivity to two well-known irreversible GABA-T inhibitors, gabaculine, and vigabatrin.

## Materials and methods

### Overexpression of recombinant human GABA-T in Expi293F™ cells

The 1503 bp human GABA-T transcript variant1 (Origene, SC127432) was amplified by PCR using nPfu-Forte (Enzynomics) and the following primers: EcoRI_ABAT (forward) 5′-aagctgtctagaGAATTCatggcctccatgttgctcgc-3′; AgeI_ABAT (reverse) 5′-acttccagatgcACCGGTttacttgaagtctgctaaga-3′. The PCR reaction mix was optimised according to the manufacturer’s protocol. After initial denaturation (95 °C for 2 min), 32 cycles (95 °C for 30 s, 58 °C for 30 s, and 72 °C for 90 s) were performed, followed by a final extension (72 °C for 5 min). The resulting PCR product was cloned into EcoRI/AgeI-cut pHR-CMV-TetO2_3C-Avi-His6_IRES-EmGFP vector (Addgene, 113888) by using an EZ-Fusion™ HT Cloning Kit (Enzynomics). The recombinant human GABA-T was overexpressed with an Expi293™ expression system (Thermo Fisher Scientific, A14635). Just before transient transfection, 5.1 × 10^8^ cells of Expi293F™ were added to 170 ml of Expi293™ Expression Medium (3 × 10^6^ cells/mL) in a 1-L flask with vent cap (Corning, CC-431147) incubated at 125 rpm, 8% CO_2_, 37 °C. For 200-ml transfection in each 1-L flask, the plasmid DNA encoding C-terminal 6xHis-tagged recombinant human GABA-T was transfected into Expi293F™ cells using ExpiFectamine™ 293 transfection kit according to manufacturer’s protocol. The incubation temperature was lowered from 37 to 32 °C immediately after enhancer addition to slow cell growth rate while further enhancing protein expression yield^32^. Overexpression of recombinant human GABA-T was monitored for 24 to 48 h via IRES-driven GFP expression in Expi293F™ cells kept in the incubator at 125 rpm, 8% CO_2_, 32 °C until transfection efficiency was reached to more than 60%. Lastly, the transfected cells were harvested and kept at −80 °C for long-term storage.

### Purification of 6xHis-tagged recombinant human GABA-T by Ni-NTA chromatography

The entire procedure must be performed at 4 °C. Approximately 6 g of cell pellet was obtained from 200 ml of cell suspension in a 1-L flask. About 36 g of cell pellet was collected from six 1-L flasks for one batch of enzyme purification. The cell pellet was resuspended in 10 volumes of 50 mM potassium pyrophosphate (K_4_P_2_O_7_) buffer at pH 8.6 containing Halt™ Protease and Phosphatase Inhibitor Cocktail, EDTA-free (Thermo Fisher Scientific, 78447). To lyse cells, sonication was carried out by using a 500 W ultrasonic processor (Sonics & Materials, Inc., VCX 500) for 60 cycles of 1 s “ON”/4 s “OFF” (1 min of sonication time) at 30% amplitude. Then, the cell lysates were centrifuged at 18,000 rpm for 30 min to remove the precipitate that might clog the chromatography column (BioRAD, 7372512). The column was filled with 4 ml of Ni-NTA resin (Qiagen, 30230) and equilibrated with 40 ml of 50 mM K_4_P_2_O_7_ buffer (pH 8.6). And all the cell lysates were applied to the column by gravity flow. After the resin was settled, the column was washed with 50 ml of 50 mM K_4_P_2_O_7_ buffer (pH 8.6) 3 times. Non-specifically bound proteins were eluted with 50 ml of 50 mM K_4_P_2_O_7_ buffer (pH 8.6) containing 10 mM imidazole. Subsequently, specifically bound 6xHis-tagged dimeric human GABA-T was eluted successively with an ascending step gradient of imidazole (125, 250, and 500 mM) in a 10-ml portion of 50 mM K_4_P_2_O_7_ buffer (pH 8.6). Finally, the eluted fractions with 125 and 250 mM imidazole were combined and concentrated with a 10 K MWCO centrifugal filter (Merk, UFC901024) before size-exclusion chromatography. The 3 C-Avi-6xHis tag at the C-terminus of the human GABA-T consists of a sequential Avi-tag and 6xHis-tag preceded by a human rhinovirus (HRV) 3 C protease site^33^. The HRV 3 C protease site allows removal of the purification tag as an option by digestion with Pierce™ HRV 3 C protease (Thermo Fisher Scientific, 88946) according to the manufacturer’s protocol. However, because small peptide tags usually do not affect protein properties^34–38^, we did not remove the purification tag which indeed did not greatly deteriorate the biological activity of the recombinant human GABA-T.

### Purification of recombinant human GABA-T homodimer by size-exclusion chromatography

The entire purification procedure was performed at 4 °C. Size-exclusion chromatography of recombinant human GABA-T homodimer was performed in a YL9100 HPLC system (Younglin Co.), equipped with an ultraviolet (UV)-visible detector. Standard (BioRAD, 1511901) or sample solutions were injected into the HPLC system through a Rheodyne injector, fitted with a 20 µL fixed loop. The separation was achieved by using Superdex 200 Increase 10/300 GL column (Cytiva, 28990944). Fifty millimolar K_4_P_2_O_7_ buffer (pH 8.6) at a flow rate of 0.5 ml/min was used as a mobile phase to transfer and separate sample proteins according to their molecular weight. The chromatograms were monitored by UV detection at a wavelength of 280 nm. The fractions were collected every minute for an hour. Lastly, the fractions containing human GABA-T homodimers were combined and concentrated with a 10 K MWCO centrifugal filter. The purified protein concentration was measured by NanoDrop One spectrophotometer (Thermo Fisher Scientific) using absorbance at 280 nm. The purified recombinant human GABA-T was stored at −80 °C for long-term storage.

### SDS-PAGE and western blot analysis

For sodium dodecyl sulfate-polyacrylamide gel electrophoresis (SDS-PAGE) in discontinuous buffer systems, we utilised discontinuous SDS-PAGE gels consisting of a large-pore stacking gel (5% acrylamide) on top of a small-pore resolving gel (10% acrylamide). The protein fractions were separated by 10% SDS-PAGE under reducing conditions followed by Coomassie Blue staining (Abcam, ab119211). For western blot analysis, the protein fractions were separated by 10% SDS-PAGE under reducing conditions and then electrophoretically transferred onto polyvinylidene difluoride (PVDF) membranes using the iBlot™ 2 Dry Blotting System (Thermo Fisher Scientific). After incubation with 5% skim milk in TBST (10 mM Tris, pH 8.0, 150 mM NaCl, 0.5% Tween 20) for 1 h at 25 °C, membranes were washed once with TBST and incubated with a 1:1000 dilution of rabbit monoclonal antibody against GABA-T (Abcam, ab108249) at 4 °C overnight. Then, membranes were washed with TBST five times and incubated with a 1:3000 dilution of horseradish peroxidase-conjugated goat anti-rabbit IgG (BioActs, RSA1221) for 1 h at 25 °C. Subsequently, blots were washed with TBST five times and developed with ECL substrate (BioRAD, 1705061). Chemiluminescent images were captured by ImageQuant LAS 500 chemiluminescence CCD camera (Cytiva).

### Isolation of bacterial SSADH from GABase

GABase (Merck, G7509) is a convenient and inexpensive source of SSADH, as it is a commercially available mixture composed of bacterial GABA-T and SSADH obtained from *Pseudomonas fluorescens*. However, GABase was not able to be used directly in our human GABA-T assay due to the presence of bacterial GABA-T. So we used the bacterial SSADH isolation method as previously described^39^, which allowed large amounts of bacterial SSADH to be isolated quickly and conveniently from GABase by irreversibly inhibiting the bacterial GABA-T activity. Five milligrams of gabaculine (Santa Cruz Biotechnology, sc-200473), a naturally occurring GABA-T irreversible inhibitor, was added to 25 ml of 1 U/mL GABase solution (reconstituted with 50 mM K_4_P_2_O_7_ buffer at pH 8.6), followed by maintaining the reaction at 4 °C for 30 min. The residual gabaculine, which also can inactivate recombinant human GABA-T, was eliminated by dialysis at 4 °C with 2 L of K_4_P_2_O_7_ buffer, changed 3 times per 9 h followed by the final dialysis overnight. The isolated bacterial SSADH was stored at −80 °C for long-term storage.

### GABA-T enzyme activity assay

All test samples were prepared at a final volume of 200 µl/well in a 96-well plate with a clear flat bottom, and the enzyme reaction was carried out at 25 °C. The final solution of the assay in Figure 3(B) consisted of SSA (0, 12.5, 25, 50, 100, and 200 µM), 2.5 mM β-NADP^+^, 1 mM 2-mercaptoethanol (2-ME), and SSADH (from 20 µL of 1 U/mL gabaculine-treated GABase) in 50 mM K_4_P_2_O_7_ buffer (pH 8.6). The final solution of the assay in Figure 3(C) consisted of GABA (0, 100 and 200 µM), 2.5 mM β-NADP^+^, 5 mM α-KG, 1 mM 2-ME and each enzymatic mixture (Mixture A: 10 µL of 1 U/mL GABase; Mixture B: 10 µL of 1 U/mL gabaculine-treated GABase; Mixture C: 2 µg of human GABA-T mixed with 10 µL of 1 U/mL gabaculine-treated GABase) in 50 mM K_4_P_2_O_7_ buffer at pH 8.6. The final solution of the assay in Figure 3(D) consisted of 200 µM GABA, 2.5 mM β-NADP^+^, 5 mM α-KG, 1 mM 2-ME, SSADH (from Mixture B) and human GABA-T at various concentrations (0.156, 0.313, 0.625, 1.25, 2.5, 5, and 10 µg/mL) in 50 mM K_4_P_2_O_7_ buffer at pH 8.6. For GABA-T inhibition assay in Figures 4(A) and 5(A), each enzymatic mixture (Mixture A or C) was preincubated with gabaculine or vigabatrin (Tocris Bioscience, 0808) at various concentrations in 50 mM K_4_P_2_O_7_ buffer (pH 8.6) containing or not containing 2-ME for 10 min at 25 °C. The final solution of the assay in Figure 4(A) consisted of gabaculine (0, 15.63, 31.25, 62.5, 125, 250, 500, and 1000 nM), 200 µM GABA, 2.5 mM β-NADP^+^, 5 mM α-KG, and each enzymatic mixture (A or C) in 50 mM K_4_P_2_O_7_ buffer (pH 8.6) containing or not containing 1 mM 2-ME. The final solution of the assay in Figure 5(A) consisted of vigabatrin (0, 0.01, 0.1, 1, 10, 100, 1000 and 10000 µM), 200 µM GABA, 2.5 mM β-NADP^+^, 5 mM α-KG, and each enzymatic mixture (A or C) in 50 mM K_4_P_2_O_7_ buffer (pH 8.6) containing or not containing 1 mM 2-ME. The change in 340 nm absorbance at 25 °C caused by the conversion of NADP^+^ to NADPH, which is proportional to GABA-T activity, was recorded every 4 min kinetically with SpectraMax iD5 Multi-Mode Microplate Reader (Molecular Devices). GABA-T activity was calculated during the linear phase of the reaction, corrected for background from blanks. All experiments were performed in triplicate and data were presented as mean ± SEM (Standard Error of Mean) or each replicate. Data plotting and regression analysis were performed with GraphPad Prism software (Version 9.1.2, GraphPad Software).

## Results and discussion

### Overexpression and purification of recombinant human GABA-T

**Figure 1.**
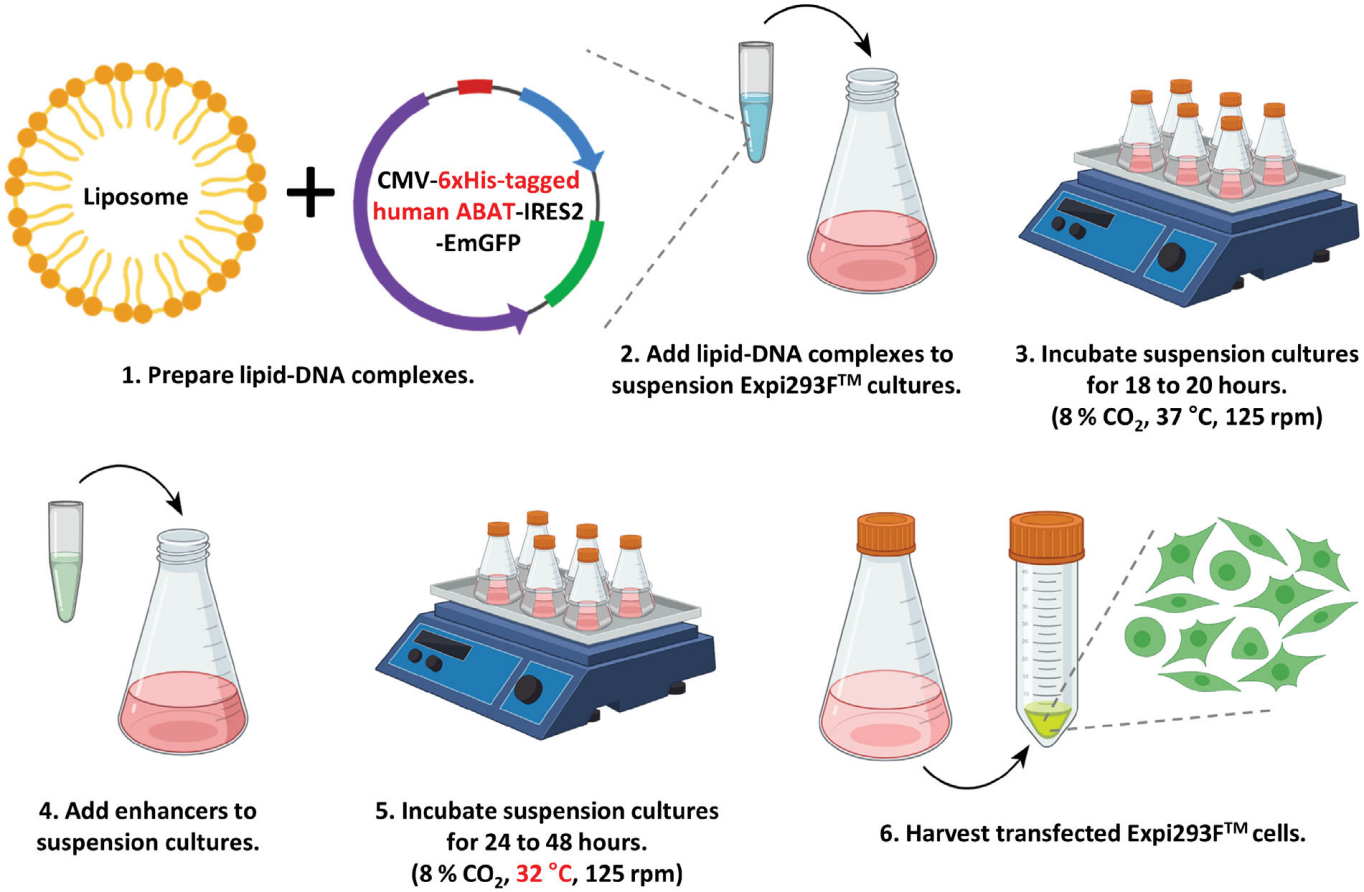
Workflow for overexpression of recombinant human GABA-T in Expi293F™ human cells.

**Figure 2.**
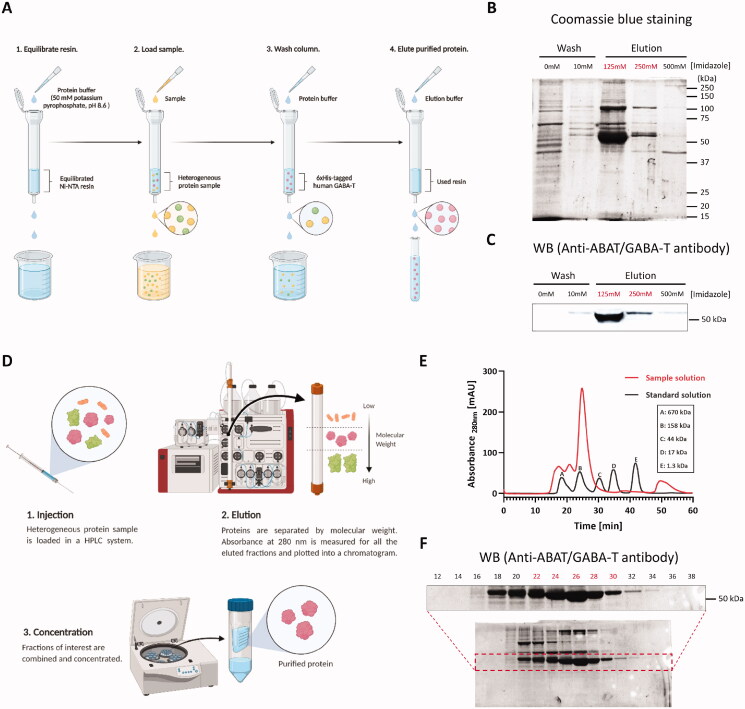
A two-step purification of 6xHis-tagged dimeric human GABA-T. (A) Workflow for purification of 6xHis-tagged human GABA-T by Ni-NTA chromatography. (B) Coomassie Blue-stained SDS-PAGE of collected protein fractions from Ni-NTA chromatography. (C) Western blot (WB) analysis of GABA-T-immunoreactive protein fractions from Ni-NTA chromatography. (D) Workflow for purification of dimeric human GABA-T by size-exclusion chromatography. (E) Chromatogram (absorbance at 280 nm) of standard solution (black) and sample solution (red). (F) Western blot (WB) analysis of GABA-T-immunoreactive and even-numbered protein fractions from size-exclusion chromatography.

To mass-produce recombinant human GABA-T with proper folding and PTMs, we developed a plasmid vector containing C-terminal 6xHis-tagged recombinant human *ABAT* and transiently overexpressed it in Expi293F™ cells by using the optimised transient transfection protocol (Figure 1). We obtained a 36 g cell pellet of the GFP-positive cells overexpressing 6xHis-tagged human GABA-T. With the cell pellet, we performed a two-step purification to obtain dimeric human GABA-T. Firstly, the 6xHis-tagged human GABA-T was purified from cell lysate via Ni-NTA purification protocol (Figure 2(A)). The elution of 6xHis-tagged human GABA-T was carried out with imidazole at concentrations of 125 and 250 mM, which was confirmed by Coomassie Blue staining and western blot analysis (Figures 2(B,C)). Secondly, we further purified the eluted fractions by size-exclusion chromatography as an optional step (Figure 2(D)), because size-exclusion chromatography should separate mammalian GABA-T, known to be present naturally as a homodimer^40^^,^^41^, from the contaminants of higher and lower molecular weights remaining after Ni-NTA chromatography, including imidazole, to ensure maximal protein purity. Given that the eluted recombinant human GABA-T exhibited a size of 53 kDa on SDS-PAGE gel under reducing conditions (Figures 2(C,F)), the molecular weight of the human GABA-T homodimer was estimated to be 106 kDa, which was actually shown in a chromatogram with the highest sample peak between standard peak B (158 kDa) and C (44 kDa) (Figure 2(E)). Finally, nine 1-min fractions from 22 to 30 min corresponding to the area under the highest sample peak were confirmed by western blot analysis (Figure 2(F)), combined, and concentrated. Taken together, we have firstly established the protocol for high-yield synthesis and purification of recombinant human GABA-T homodimer by using the human cell line, yielding 1.2 mg of recombinant human GABA-T from 36 g of cell pellet for one batch.

### Validation and optimisation of human GABA-T activity assay

**Figure 3.**
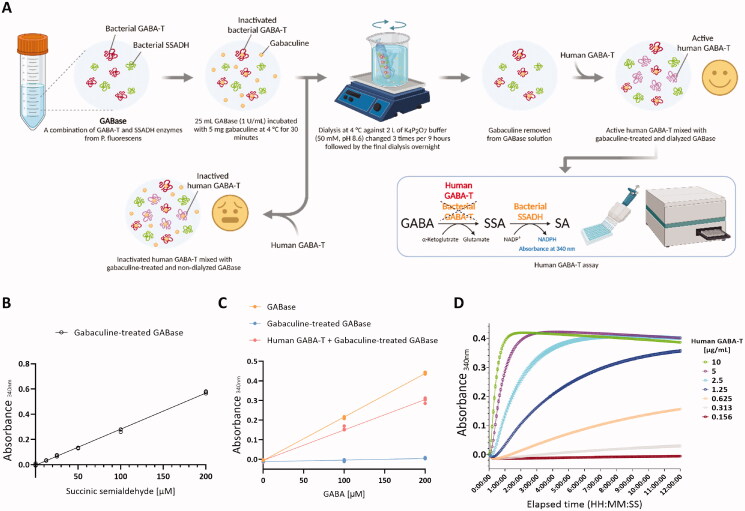
Validation and optimisation of human GABA-T activity assay. (A) Workflow for preparing human GABA-T activity assay consisting of the purified recombinant human GABA-T and bacterial SSADH isolated from GABase. (B) A plot of bacterial SSADH activity as a function of SSA concentration for validating the activity of bacterial SSADH in gabaculine-treated GABase. (C) Plots of the activity of recombinant human GABA-T, bacterial GABA-T in GABase, and bacterial GABA-T in gabaculine-treated GABase as a function of GABA concentration for validating the activity of recombinant human GABA-T in comparison with that of bacterial GABA-T in GABase and bacterial GABA-T in gabaculine-treated GABase. (D) Time course plots of the activity of recombinant human GABA-T at different concentrations for optimising recombinant human GABA-T concentration and assay timing.

To establish a coupled assay system consisting of the purified recombinant human GABA-T and NADP^+^-dependent bacterial SSADH isolated from GABase, we validated the activity of each of the two enzymes step by step. Firstly, we separately isolated bacterial SSADH from the commercially available GABase. The isolated bacterial SSADH was mixed with the purified human GABA-T for the conversion of GABA and α-KG to SSA and glutamate, followed by the conversion of SSA and NADP^+^ to SA and NADPH, which can be detected by the changes in absorbance at 340 nm (Figure 3(A)). To confirm the activity of bacterial SSADH in gabaculine-treated GABase, we performed a standard calibration with SSA at different concentrations and obtained a linear relationship between absorbance at 340 nm and SSA concentration, indicating that bacterial SSADH in gabaculine-treated GABase showed normal activity (Figure 3(B)). Next, we compared the activity of bacterial GABA-T in GABase, bacterial GABA-T in gabaculine-treated GABase, and the purified recombinant human GABA-T. GABA degradation-dependent NADPH synthesis was completely impaired by gabaculine treatment in GABase and mostly recovered by replacing the inactivated bacterial GABA-T with the purified recombinant human GABA-T (Figure 3(C)). Finally, the optimal concentration of the recombinant human GABA-T and assay timing was determined by real-time measurement of human GABA-T activity at different concentrations (Figure 3(D)). Ideally, human GABA-T activity should be measured during the linear phase of the reaction. The reaction progress curve in cyan denotes an optimal human GABA-T concentration (2.5 µg/mL), as it is linear for a sufficient duration (2 h) to be assayed and has a sufficient signal strength (Figure 3(D)). We also calculated the specific enzyme activity of human GABA-T, which is an important measure of enzyme purity. One enzyme unit (1 U = 1 µmol/min) is defined as the amount of the enzyme that catalyses the conversion of 1 µmol of the substrate into product per minute under the specified conditions of the assay method^42^. Human GABA-T at a concentration of 2.5 µg/mL converted 0.00125 µmol of the substrate to product per minute per mL (pH 8.6, 25 °C), which is defined as enzyme activity (0.00125 U/mL). We divided the enzyme activity by the concentration of human GABA-T to get the specific enzyme activity (0.5 U/mg). Taken together, we established the human GABA-T (0.5 U/mg) activity assay protocol with the optimal concentration (2.5 µg/mL) and reaction time (2 h). Under this protocol, 1.2 mg of recombinant human GABA-T from 36 g of cell pellet is sufficient for 2400 assays at 200 µL/assay in 96-well plates, allowing for a high-throughput drug screening.

### Direct comparison of the differences between human and bacterial GABA-T in sensitivity to two irreversible inhibitors

Mammalian GABA-T is a homodimer with the two independent active sites positioned near the interface between the two monomers. Each monomer is complexed to pyridoxal 5′-phosphate (PLP), which is a coenzyme and covalently bound to Lys-329 at the active site of porcine GABA-T by a Schiff base (an internal aldimine bond)^41^. PLP-dependent GABA-T operates via a “ping-pong” mechanism, two half-reactions completing a full transamination cycle^26^^,^^43^. It also has been reported that the Cys-321 residue at the active site of human GABA-T is involved in the formation of a disulphide link between two monomers of GABA-T, which is supported by the evidence that the wild type GABA-T, but not the Cys321 mutants, exists as a homodimer that is dissociable into monomers by 2-mercaptoethanol (2-ME)^44^. In contrast, there have been several reports that the bacterial GABA-T exists in multimeric forms of higher-order than dimers^45^^,^^46^.

**Figure 4.**
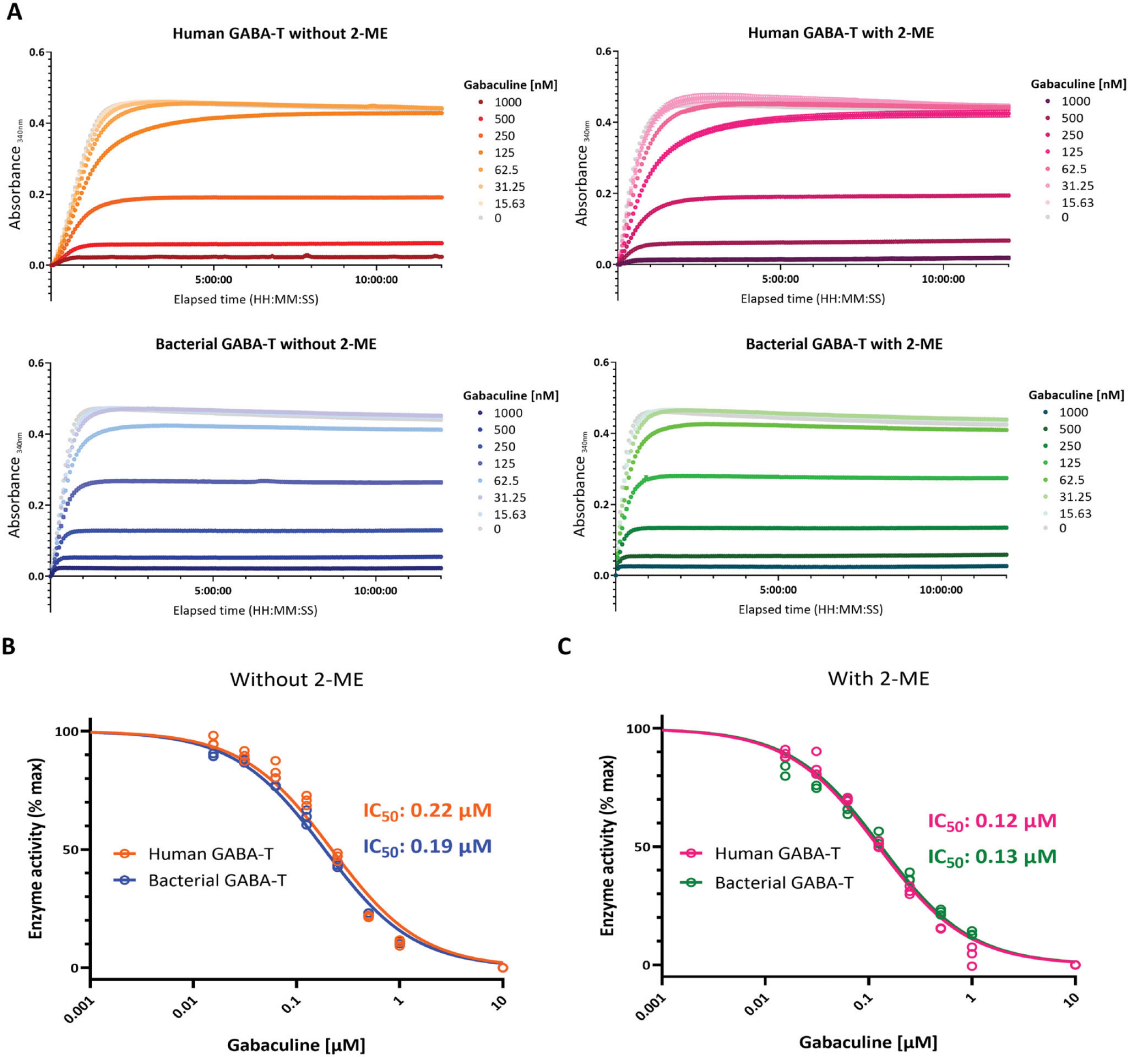
Comparisons of the inhibitory effects of gabaculine on human and bacterial GABA-T under non-reducing or reducing conditions. (A) Comparisons of pharmacological profiles between human and bacterial GABA-T with gabaculine at different concentrations in the absence or presence of 2-ME. (B) Dose-response curves of human and bacterial GABA-T inhibition by gabaculine in the absence of 2-ME. (C) Dose-response curves of human and bacterial GABA-T inhibition by gabaculine in the presence of 2-ME.

**Figure 5.**
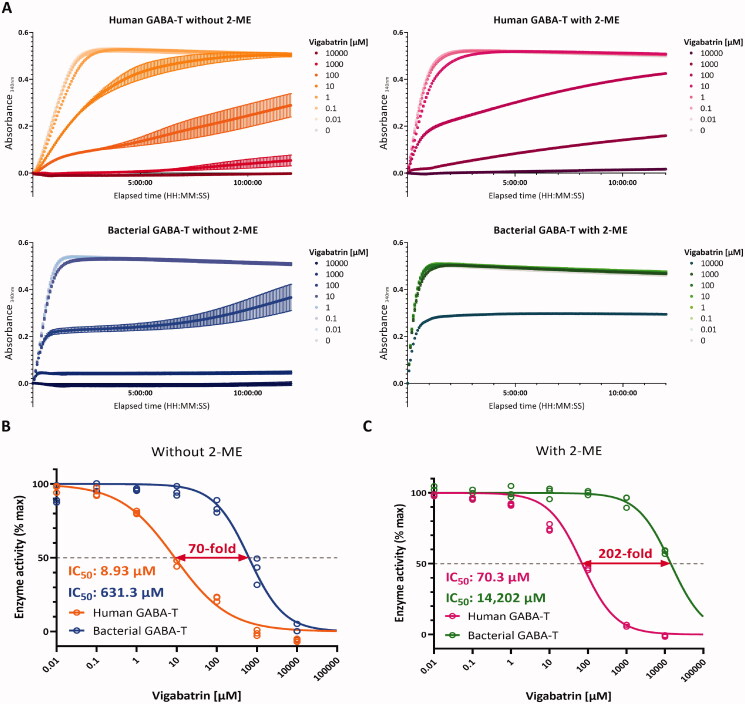
Comparisons of the inhibitory effects of vigabatrin on human and bacterial GABA-T under non-reducing and reducing conditions. (A) Comparisons of pharmacological profiles between human and bacterial GABA-T with vigabatrin at different concentrations in the absence or presence of 2-ME. (B) Dose-response curves of human and bacterial GABA-T inhibition by vigabatrin in the absence of 2-ME. (C) Dose-response curves of human and bacterial GABA-T inhibition by vigabatrin in the presence of 2-ME.

The mechanisms of irreversible inhibition of gabaculine and vigabatrin are well-known. The irreversible inhibition is observed when gabaculine is covalently linked to PLP and this adduct is bound tightly to the active site of GABA-T^47^. Similarly, vigabatrin forms a covalent ternary adduct with the active site Lys-329 and PLP cofactor to irreversibly inhibit GABA-T^41^. However, the values of half-maximal inhibitory concentration (IC_50_) of gabaculine and vigabatrin have not been determined with human GABA-T, due to the lack of availability. To test whether IC_50_ values of gabaculine and vigabatrin for GABA-T differ by the species and protein quaternary structure, we recorded kinetic profiles of human and bacterial GABA-T reactions with gabaculine or vigabatrin at different concentrations in the absence or presence of 2-ME (Figures 4(A) and 5(A)). Dose-response curves were plotted by using the data from the linear initial phase of the reactions. IC_50_ values of gabaculine for human GABA-T and bacterial GABA-T in the absence of 2-ME (multimeric form) were 0.22 and 0.19 µM, respectively (Figure 4(B)). IC_50_ values of gabaculine for human GABA-T and bacterial GABA-T in the presence of 2-ME (monomeric form) were similar as well; 0.12 and 0.13 µM, respectively (Figure 4(C)). On the other hand, in the absence of 2-ME, IC_50_ of vigabatrin for bacterial GABA-T (631.3 µM) was higher than for human GABA-T (8.93 µM) (Figure 5(B)), indicating that the human GABA-T is more sensitive to vigabatrin than the bacterial GABA-T. Likewise, in the presence of 2-ME, IC_50_ of vigabatrin for bacterial GABA-T (14,202 µM) was higher than for human GABA-T (70.3 µM) (Figure 5(C)). These results indicate that although the human and bacterial GABA-T showed similar sensitivity to gabaculine, the human GABA-T in homodimeric form showed 70-fold higher sensitivity to vigabatrin than the bacterial GABA-T in multimeric form. In addition, the difference in sensitivity to vigabatrin between the human and bacterial GABA-T significantly increased from 70-fold in the multimeric form to 202-fold in the monomeric form. Taken together, the human and bacterial GABA-T have profoundly different characteristics, such that the human GABA-T cannot be substituted with the bacterial GABA-T, especially when searching for GABA-T modulators for human use.

## Conclusions

Our results are consistent with the findings of previous studies implicating that the difference between human GABA-T and GABA-T from other species is obvious in the irreversible inhibitory effect of vigabatrin towards GABA-T. This can be presumably supported by the evidence that *E. coli* GABA-T does not have an iron-sulfur cluster of yet unknown function that is present at the centre of the mammalian GABA-T homodimer in the vicinity of the PLP binding site^41^^,^^45^. Moreover, human GABA-T is immunologically distinct from other mammalian enzymes^48^ and more sensitive to vigabatrin than GABA-T isolated from bovine brain^30^, even though the amino acid sequence of bovine GABA-T shows 96% identity with human GABA-T^31^. Therefore, IC_50_ values of CPP-115 and OV329 for human GABA-T and the crystal structure of human GABA-T inactivated by CPP-115 or OV329 should also be determined with our newly developed protocol in the future. In conclusion, all of these rationales can be put forward for this study providing a scalable, efficient, and high-yield synthesis and purification protocol for recombinant human GABA-T which can be a readily available source to facilitate further biochemical studies and development of more effective GABA-T modulators for the treatment of human CNS diseases.
